# Molecular Detection and Isolation of Lumpy Skin Disease Virus during an Outbreak in West Hararghe Zone, Eastern Ethiopia

**DOI:** 10.1155/2024/9487970

**Published:** 2024-02-29

**Authors:** Umer Seid Geletu, Ahmedin Abdurehman Musa, Munera Ahmednur Usmael, Melaku Sombo Keno

**Affiliations:** ^1^Department of Animal Science, College of Agriculture, Oda Bultum University, P.O. Box 226, Chiro, Ethiopia; ^2^Oromia Bureau Livestock and Fishery Resources, West Hararghe Zone, P.O. Box 226, Wereda, Chiro, Ethiopia; ^3^National Animal Health Diagnostic and Investigation Center, Sebeta, Ethiopia

## Abstract

Lumpy skin disease (LSD) is a highly contagious viral disease that causes significant economic losses in cattle populations globally. This study aimed to isolate and detect the LSD virus responsible for an outbreak in selected areas (Daaroo Labuu, Hawwii Guddina, and Gumbi Bordede district) of the West Hararghe Zone in Ethiopia between January 2020 and December 2021. Out of the 625 animals examined for the presence of LSD, only 73 animals showed clinical signs, and skin scrapes were collected from these animals for further analysis. Among those, 12 animals (1.9%) succumbed to the disease. Skin biopsy samples from 45 animals displaying clinical signs of LSD were inoculated in Vero cell lines because of limited equipment. After three blind passages, all samples developed cytopathic effects (CPEs). The presence of the LSD virus was confirmed using real-time PCR. Conventional PCR detected LSDV in 47 (64.4%) of the skin scrap samples, while high-resolution melt qPCR detected it in 49 (67.1%) samples. The study revealed a morbidity rate of 11.68%, a mortality rate of 1.92%, and a case fatality rate of 16.44% based on clinical data. The findings suggest that LSD causes significant economic losses, even in vaccinated animals prior to an outbreak. To effectively control and eradicate LSD, the government should develop new strategic policies. Community awareness campaigns are necessary to improve vector control measures and drainage systems. In addition, the present vaccination policy and strategy should be re-evaluated for effectiveness. This study focused on a specific region and timeframe, limiting generalizability. Factors such as environmental conditions and management practices were not extensively explored. Similar studies should be conducted in different regions to assess the prevalence and genetic diversity of LSDV. The effectiveness of control measures and vaccination strategies should be investigated. The impact of environmental factors and management practices on LSD transmission and disease severity warrants further exploration. This study provides insights into the detection and isolation of the LSD virus during an outbreak in the West Hararghe Zone of Ethiopia. The results highlight the need for continued surveillance and monitoring of emerging infectious diseases in the region. Furthermore, the importance of using molecular methods for detecting and characterizing viral outbreaks in livestock populations is emphasized.

## 1. Introduction

Lumpy skin disease (LSD) is an increasingly prevalent disease that significantly impacts economic losses. It is a viral disease of cattle caused by the lumpy skin disease virus (LSDV) a member of Poxviridae of the *Capri poxvirus* genus, and the disease often occurs in epidemics. This disease originated in Zambia, then it spread to most African countries in 1929, Middle Eastern countries, and also in European countries. This disease was able to be found in a highly diverse ecological zone [1, 2]. In Ethiopia, lumpy skin disease was first found in the northwestern part of Gojjam and Gondar of the country in 1981, then introduced in the western part of Wollega in 1982 from Sudan and in the central part of Shewa in 1983. Currently, this disease is spread all over the country [3]. Because of the inability to control movements of cattle, grazing and watering in common areas, pastoralism, and the poor handling of diseased animals with the late detection of animal disease has contributed to the transmission of the lumpy skin disease virus in Ethiopia [2, 3].

The lumpy skin disease (LSD) virus is a member of the Capripoxvirus genus, a group of large, double-stranded DNA viruses that infect animals. It is specifically classified as a poxvirus, belonging to the family Poxviridae. The LSD virus primarily affects cattle and is characterized by the formation of distinctive skin lesions. Infected animals develop raised nodules on their skin, which gradually enlarge and become firm, resulting in the characteristic lumpy appearance. In addition to the visible skin lesions, LSD virus infection can cause systemic symptoms such as fever, reduced appetite, and general malaise. The virus is highly contagious and can spread rapidly among cattle herds through direct contact, insect vectors, or contaminated materials. The economic impact of LSD virus infection is significant, as it can lead to reduced milk production, weight loss, decreased fertility, and even death in severe cases. Effective management strategies, including vaccination, vector control, and strict biosecurity measures, are crucial for preventing and controlling the spread of the LSD virus in cattle populations [4, 5]. In contrast, LSD is endemic in Africa, and outbreaks have also been reported in parts of Europe, including Greece, the Middle East, and the regions bordering Egypt [6]. Lumpy skin disease poses an imminent threat to numerous African countries and has recently expanded its presence to various regions in Europe, the Middle East, South Asia, and Southeast Asia [7].

One of Ethiopia's limitations on livestock output is cattle illness. Because of the endemic prevalence of LSD, the sheep, goat, and goat pox viruses, and other ruminant diseases, the nation is having significant issues exporting live ruminants and their products. In addition, the loss of meat and milk production as well as the subpar quality of skin and hides have a negative impact on the growth of the national economy. Recognized causes of the seasonality of vector-borne disease include seasonal fluctuations in vector abundance. Due to an increase in the vector's population during the rainy season, LSD epidemics are more common than during the dry and cold weather seasons. LSD outbreaks vary seasonally in Ethiopia, most frequently occurring between September and December 7. The heavy rains, the introduction of many vectors, and a poor degree of herd immunity have all been consistently related to a resurgence of LSD. Knowing the seasonal variations of LSD outbreaks in great detail is crucial for comprehending the dynamic nature of this infectious disease and for creating the best control and preventative measures [8].

The LSD outbreak has happened as a major epidemic in different regions of Ethiopia such as the Amhara and Oromia regions in 2000/2001, the Oromia and SNNP regions in 2003/2004, and the Tigray, Amhara, and Benishangul regions in 2006/2007. LSD is a notifiable disease and is, therefore, subjected to continuous surveillance. Even if there have been different studies undertaken on the investigation of LSD outbreaks in different areas of the country, no study has been done in the Western Hararghe Zone. Thus, the purpose of this study was to investigate the emergence of lumpy skin disease and molecularly identify the LSD virus.

## 2. Materials and Methods

### 2.1. Description of the Study Area

**Figure 1:**
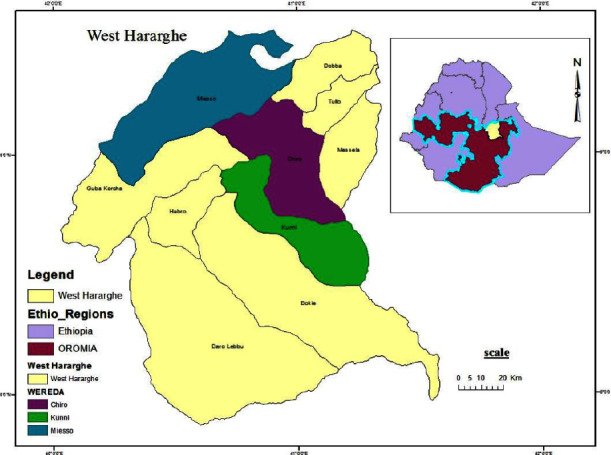
Study area map (Umer Seid Geletu).

In response to concerns about outbreaks of lumpy skin disease (LSD), the study was carried out in a particular region in the West Hararghe Zone, Ethiopia, between January 2020 and December 2021. West Hararghe Zone is 317 kilometres from Addis Ababa in the eastern region of Ethiopia (Figure 1). The research region, which has an elevation range of 1200 to 3600 meters above sea level, is located between the latitudes of 70° 52′ 15″ and 90° 28′ 43″ N and the longitudes of 400° 03′ 33″ and 400° 34′ 13″ E. Three agro-climatic zones—highland (Dega), midland (Weina Dega), and lowland (Kola)—define the region. Kola occupies 49.51 percent of the territory, Dega 12.4 percent, and Weina Dega 38%. Belgi/Badhesa (February–April) and Ganna (June–September) are the two rainy seasons in the region. The region's typical annual rainfall is between 650 and 1500 millimetres, while the average temperature is between 20.5 and 24 degrees Celsius [9].

The study area's several rainy seasons and variety of agro-climatic zones make it an ideal environment for livestock. However, these circumstances also raise the possibility of illness outbreaks, including LSD. The goal of the study was to better understand the local LSD epidemiology and create efficient control measures. The research area's location and climate offer important insights into the dynamics of LSD transmission in related environments, which can help with the creation of efficient disease management initiatives [9].

### 2.2. Study Population

This study focused on cattle that had skin lesions that were clinically indicative of pox. Based on information gathered from several districts and focal people at local veterinary clinics, active outbreaks were looked at. All cattle in the age group, whether they were local zebu or alien cattle, were taken into consideration for the study on the farms. In response to outbreaks of lumpy skin disease (LSD), all investigations were carried out. Semistructured inquiries were used to gather primary information during the outbreak investigations from farm owners and veterinarians in charge of overseeing the farms. The overall number of cattle, the number of affected livestock, the number of dead cattle, and the clinical findings were the main topics of the questioning. The outcomes of the clinical examination were documented in a preplanned style.

Semistructured questions were used to acquire reliable and consistent data from the farms. Clinical examination results were recorded in a uniform format to guarantee consistency and make analysis simple. These steps were essential in order to accurately assess the scope and severity of the LSD outbreaks in the region.

### 2.3. Study Design

The study's goal was to look into a lumpy skin disease (LSD) outbreak in cattle that occurred during a two-year period, from January 2020 to December 2021, in a particular area. Specifically chosen houses with cattle were included in the study population based on epidemic reports from the district animal health services office. Clinical LSD symptoms such as skin nodules, swollen lymph nodes, lacrimation, lameness, and fever were checked in cattle. The age, sex, breed, and immunization records of the animals were also recorded. The incidence of the disease and fatalities in the afflicted cattle herd was evaluated using a cross-sectional approach in the study. Observation and interviews with cattle owners and experts in animal health were used as data collection techniques. To calculate the disease's incidence, mortality, and case fatality rates in the affected population, descriptive statistics were used to assess the data that had been obtained. In general, the study sought to shed light on the epidemiology of LSD epidemics in the area and inform the creation of efficient disease control policies.

### 2.4. Sample Collection and Transportation

In order to identify the virus that causes lumpy skin disease (LSD), samples were taken from skin nodules on 73 representative cattle who had the disease's severe clinical symptoms. Skin nodules were aseptically collected according aseptically collected by WOAH [10] guidelines by washing, disinfecting, and shaving the area to remove any hairs. A sterile universal container containing phosphate-buffered saline (PBS) at a pH of 7.2–7.6 and antibiotics (gentamicin) was sterilized before being filled with about 2–5 g of samples to be used as a virus transport medium (VTM) [17, 18].

According to the WOAH terrestrial guideline (2017), a purposive sampling method was used to gather the samples from the LSD outbreak area. A thorough medical examination was performed prior to taking samples from the affected animals. Each representative cattle had two skin biopsy samples from cutaneous nodules taken aseptically by washing and disinfecting the region with a sterile scalpel blade.

To keep the cold chain system intact, the tissue samples were delivered to the National Animal Health Diagnosis and Investigation Center (NAHDIC) in sterilized universal bottles containing tryptase phosphate broth. After that, the tissue samples were kept at −20°C until they were prepared for further examination. The integrity of the samples was guaranteed, and the risk of contamination during the isolation process was significantly reduced by the use of aseptic procedures during sample collection and transport. Utilizing a virus transport medium and a cold chain system helped keep the virus viable while being transported. These precautions were essential for the virus's successful isolation and subsequent characterizations.

### 2.5. Laboratory Diagnosis

#### 2.5.1. Virus Isolation

To prepare the biopsy samples for virus isolation, they were first thawed at room temperature and washed three times in sterile phosphate-buffered saline (PBS, pH 7.2). Approximately 1 gram of the washed tissue sample was then mixed with 9 ml of sterile PBS containing 0.1% gentamicin antibiotic (Sigma-Aldrich, Germany) and ground using a sterile mortar and pestle. The resulting tissue suspension was centrifuged at 600 x g for 15 minutes, and the supernatant was filtered through a 0.45 *μ*m pore size membrane (Millipore, USA). The filtered supernatant was then inoculated onto a monolayer of Vero cells in 25 cm^2^ tissue culture flasks, and the flasks were incubated at 37°C for 1 hour to allow for virus adsorption. Next, 9 ml of Glasgow minimum essential medium (GMEM, Sigma-Aldrich) containing 0.1% gentamicin and 2% fetal calf serum (Sigma-Aldrich) was added to the flasks. The inoculated flasks were then incubated at 37°C in a humidified incubator with 5% CO2. The Vero cells were monitored daily for 14 days using an inverted microscope for evidence of virus-induced cytopathic effects (CPEs). Finally, the cells were frozen at −80°C for further analysis. This method of virus isolation allowed for the detection and characterization of the virus in the biopsy samples. The use of Vero cells as a monolayer provides a suitable environment for virus replication and propagation. The monitoring of the cells for CPEs provides evidence of virus infection and can inform the development of effective control strategies for the disease [8, 18].

#### 2.5.2. DNA Extraction

To extract the DNA of lumpy skin disease virus (LSDV) from processed tissue samples, the study used the QIAmp Viral DNA Mini Kit (QIAGEN, Germany) following the manufacturer's instructions at the NAHDIC molecular biology laboratory. First, 20 *μ*l of proteinase K was added to all tubes according to the sample size, and then 200 *μ*l of collected supernatants were added. Next, 200 *μ*l of AL buffer was added and mixed together using a vortex mixer. The mixture was then incubated at 56°C for 30 minutes in a water bath and briefly centrifuged. To bind the nucleic acid on the mini spin column, 200 *μ*l of ethanol (96–100%) was added to the mixture, which was then mixed using a vortex mixer for 15 seconds and briefly centrifuged. The mixture was then transferred to the QIAamp mini spin column and centrifuged at 6000 × g for 1 minute. The spin column containing the DNA was then transferred to a new 2 ml collection tube. To purify the DNA, the first washing buffer, 500 *μ*l AW1, was added to the spin column and centrifuged at 6000 × g for 1 minute. Next, 500 *μ*l of the second washing buffer, AW2, was added and centrifuged at 14000 rpm for 3 minutes. The filtrate was then discarded, and this step was repeated for 1 minute without adding any buffer. The mini spin column was then transferred to a microcentrifuge tube, and 200 *μ*l of AE elution buffer was added. The mixture was incubated at room temperature for 1–5 minutes to increase the yield of DNA and then eluted by centrifugation at 6000 × g for 1 minute using the fifth edition QiAamp DNA extraction protocol from [13]. The use of the QIAmp Viral DNA Mini Kit allowed for efficient and reliable extraction of DNA from the processed tissue samples. The purified DNA can be used for downstream applications, such as PCR analysis, to detect the presence of LSDV in the samples.

#### 2.5.3. Conventional PCR

The study used PCR procedures according to Wallace et al. [14] to detect the presence of lumpy skin disease virus (LSDV) in the extracted DNA samples. The primer sets used targeted the p32 gene, which codes for the viral attachment protein. The forward primer, 5′-TTTCCTGATTTTTCTTACTAT-3′, and the reverse primer, 5′-AAATTATATACGTAAATAAC-3′, were designed by Zeedan et al. [15]. In this study, DNA extraction was performed following the protocol described by Wallace et al. [14]. A 0.5 mL volume of the infected sample suspension was processed by adding 20 *μ*L of proteinase K (with a final concentration of 100 *μ*g mL^−1^), followed by incubation at 56°C for 2 hours. Subsequently, 100 *μ*L of phenol : chloroform : isoamyl alcohol (25 : 24 : 1) was added, and after thorough mixing and centrifugation at 13,000 rpm for 5 minutes, the upper aqueous layer containing the DNA was transferred to a clean microcentrifuge tube. For DNA precipitation, 2.5 volumes of absolute ethanol and 1/10 volume of 5 mol L^−1^ sodium acetate (pH 5.3) were added and mixed thoroughly. The mixture was then incubated at −20°C overnight, followed by centrifugation at high speed (13,000 rpm) for 15 minutes to pellet the DNA. The pellet was washed once with 70% ethanol, centrifuged at 12,000 rpm for 10 minutes, air-dried, and finally resuspended in 50 *μ*L of T/E buffer (PureLinkTM). Negative control samples, consisting of normal noninfected samples, were included to ensure the absence of contamination during the DNA extraction process. It is important to note that the specific volumes, concentrations, and incubation times may need to be optimized based on the experimental requirements and sample characteristics.

#### 2.5.4. Agarose Gel Electrophoresis

To confirm the presence of DNA in the extracted specimens, the amplified DNA was analyzed using agarose gel electrophoresis, following the method described by Mangana-Vougiouka et al. [16] with some modifications. The amplified products were analyzed using a molecular marker, Gene RulerTM 100 bp DNA ladder (Fermentas, Germany), on 2% agarose gels prepared in Tris/acetate/EDTA (TAE) buffer with a 10 mg/ml ETDM-bromide strain. To load the samples, 20 *μ*l of PCR product was mixed with 4 *μ*l loading buffer and loaded into wells in the gel. The gel was then run at 100 volts for approximately 60 minutes in parallel with the DNA molecular weight marker in the electrophoresis apparatus until the DNA samples had migrated a sufficient distance through the gel. To visualize the DNA bands, a UV transilluminator was used at a wavelength of 590 nm. Positive results were confirmed based on the size of the bands formed on the agarose gel. A PCR result was considered positive for LSDV-DNA when a 192 bp band was observed. This method of confirming the presence of DNA allowed for accurate and efficient analysis of the amplified products. The findings can contribute to the understanding of the epidemiology of LSD outbreaks and inform the development of effective control strategies for the disease [17].

#### 2.5.5. Real-Time PCR (qPCR)

A TaqMan-based quantitative reverse transcription polymerase chain reaction (qRT-PCR) analysis was performed using a pair of specific primers and a probe targeting the p32 gene. The primers and probe were synthesized by Sangon Biotech Co., Ltd. (Shanghai). The qRT-PCR amplifications were conducted in a 25 *μ*l reaction system, which included 5 *μ*l of extracted sample nucleic acid or template controls and 20 *μ*l of a prepared master mix. The PCR master mix consisted of 12.5 *μ*l of 2× Taq MasterMix (Vazyme, Nanjing, China), 1 *μ*l of each primer, 0.5 *μ*l of the probe, and 5 *μ*l of RNase-free ddH_2_O. The amplification process involved the following thermocycling conditions: an initial step of 50°C for 2 minutes, followed by 95°C for 5 minutes, and then 40 cycles of amplification (95°C for 15 seconds and 58°C for 15 seconds). The FAM (5-carboxyfluorescein) signal was collected at 58°C during each cycle for quantitative analysis. The qRT-PCR reaction was carried out using a Quant Studio 5 PCR instrument (Thermofisher, USA), and samples with a CT value of less than 40 were considered positive.

#### 2.5.6. Data Analysis

During the outbreak investigation, data on the number of animals at risk and the number of deaths were collected and entered into a Microsoft Excel spreadsheet. The data were organized based on region and site of collection. To determine the impact of the outbreak on the affected cattle population, the study calculated the percentage of mortality by dividing the number of deaths by the number of animals at risk and multiplying by 100. The fatality rate was also calculated by dividing the number of deaths by the number of sick animals and multiplying by 100. The use of Microsoft Excel allowed for efficient organization and management of the data, making it easier to calculate and interpret the morbidity and mortality rates. These rates serve as important indicators of the severity of the outbreak and can inform the development of effective control strategies for the disease.

## 3. Result

### 3.1. Outbreak Investigation

This research explored outbreaks of lumpy skin disease (LSD) that occurred in the West Hararghe Zone from January 2020 through December 2021. The study area included three districts: Daaroo Labuu, Hawwii Guddina, and Gumbi Bordede. Out of the 625 animals examined for the presence of LSD, only 73 animals showed clinical signs, and skin scrapes were collected from these animals for further analysis.

**Figure 2:**
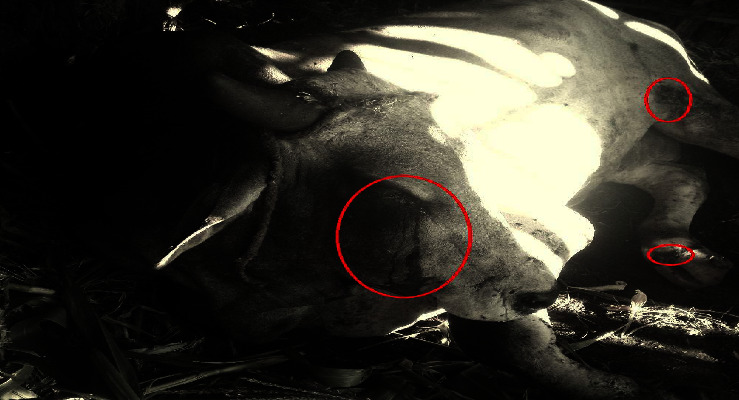
According to the authors, the features that define lumpy skin disease include necrotized skin and excessive tearing (lacrimation).

**Figure 3:**
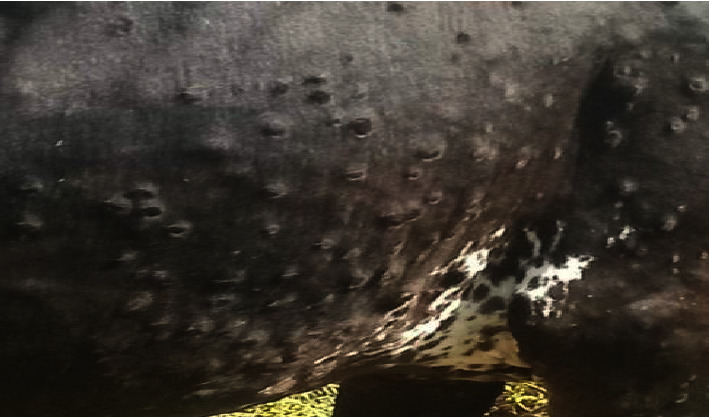
A skin nodule is one of the characteristic clinical signs commonly observed in cases of lumpy skin disease (LSD).

The most commonly observed clinical signs of LSD in the affected animals were skin nodules, necrotized nodules, depression, lacrimation (Figure 2 and 3), in appetence, salivation, nasal discharge, enlarged peripheral lymph nodes, and lameness (Figures 2 and 3). These clinical signs are consistent with previous reports of LSD in other regions and highlight the severity of the disease in the study area.

The collection of skin scrapes from animals showing clinical signs of LSD allowed for the isolation and identification of the virus responsible for the outbreaks. The information gathered from this study is critical for understanding the epidemiology of LSD in the region and developing effective control strategies. The data can also inform the development of surveillance programs to detect and monitor LSD outbreaks in the future.

During the study period, lumpy skin disease (LSD) outbreaks in cattle resulted in significant morbidity and mortality. Data analysis indicated that the morbidity rate, mortality rate, and case fatality rate were 11.68%, 1.92%, and 16.44%, respectively, in the study area (Table 1). The high case fatality rate highlights the severity of LSD in the affected animals.

The current study found that the Hawwii Guddina district had the highest morbidity rate among the three districts studied. However, the mortality and case fatality rates in this district were relatively lower compared to the other districts (Table 1).

The high morbidity and case fatality rates observed in the study area suggest that LSD is a significant threat to cattle in the region. The data obtained from this study can help inform the development of effective control strategies for LSD. The identification of districts with higher morbidity rates can also guide the implementation of targeted intervention measures to prevent further spread of the disease (Table 2).

### 3.2. Molecular Detection

Out of the 73 skin biopsy samples that showed characteristic poxvirus lesions, only 45 samples were inoculated in Vero culture. After three blind passages, all 45 samples developed cytopathic effects (CPEs) characterized by the aggregation of dead cells and the destruction of monolayers.

The successful isolation of the virus responsible for LSD in the Vero culture is an important step towards understanding the characteristics of the virus and developing effective control measures. The CPEs observed in the Vero culture are consistent with previous reports of LSD virus propagation in cell cultures. The use of blind passages ensured the purity of the virus and minimized the risk of contamination during isolation. The isolated virus can be further characterized using a range of molecular and serological techniques, which will provide valuable information on the genetic and antigenic properties of the virus.

### 3.3. Polymerase Chain Reaction

Polymerase chain reaction (PCR) was used to target the p32 gene region of the virus isolated from the collected skin biopsy samples. Analysis of the PCR products using 2% agarose gel electrophoresis showed a fragment size of 192 bp, which is consistent with the LSD virus.

**Figure 4:**
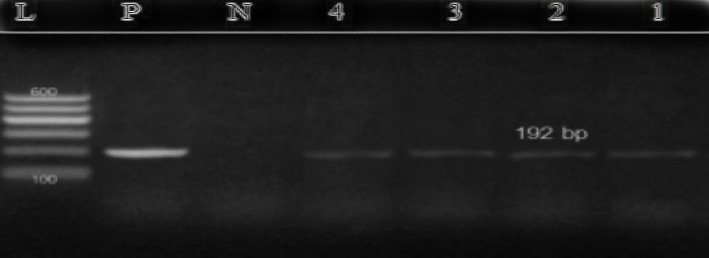
The amplified p32 gene was visualized on an agarose gel electrophoresis, showing a band of 192 base pairs. The samples on the gel are labeled as follows: L represents the DNA ladder, P represents the positive control, N represents the negative control, and 1, 2, 3, and 4 represent the positive samples.

In the current study, conventional PCR was used to test skin scrapings from cattle for the presence of the LSD virus. The results showed that 64.4% (47/73) of the samples were positive for LSDV using a specific primer set that amplified a 192 bp DNA fragment, which is the expected amplification product size for LSDV. The molecular weight of the PCR products from the positive samples was 192 bp, which matches the expected amplification product size from the reference LSDV (Figure 4).

The use of PCR allowed for the rapid and accurate detection of LSDV in the skin biopsy samples. The high rate of positivity observed in the study suggests that LSD is widespread in the study area and highlights the need for effective control measures to prevent the further spread of the disease. The molecular characterization of the LSDV isolates can also provide valuable insights into the genetic diversity of the virus and inform the development of effective diagnostic and control strategies.

### 3.4. Real Time Polymerase Chain Reaction

The presence of specific Ct curve values of DNA templates isolated from the Vero cell line with pock lesions and skin biopsy samples from diseased animals indicated the presence of the lumpy skin disease virus (LSDV). The Ct values of these samples were above the threshold curve and were almost the same as the Ct values of the positive control of the LSDV DNA template.

A potent method for identifying genetic variants, mutations, and polymorphisms in DNA samples is high-resolution melt (HRM) analysis. A specified section of DNA is amplified using polymerase chain reaction (PCR), and the amplified DNA is then subjected to a series of temperature cycles that cause the DNA to denature. A high-resolution melting equipment can track this shift in real-time as the fluorescence of a dye linked to the DNA changes as the DNA strands denature. HRM analysis may identify various genotypes and identify minute differences in DNA sequences by examining the pattern of melting curves. With applications in research, clinical diagnostics, and forensic science, HRM analysis provides a quick, accurate, and reasonably priced approach for genetic analysis. High-resolution melt (HRM) analysis was used for the qPCR detection of LSDV in skin scrapings, which showed a positivity rate of 67.1% (49/73). The HRM analysis is a sensitive method for detecting DNA sequence variations, and the high positivity rate in the skin scrapings suggests that LSDV is widespread in the study area^23^.

The use of multiple diagnostic methods, including PCR, Ct curve analysis, and HRM analysis, allowed for the rapid and accurate detection of LSDV in the study samples. The high positivity rate observed in the skin scrapings highlights the need for effective control measures to prevent further spread of the disease. The molecular characterization of the LSDV isolates can also provide valuable insights into the genetic diversity of the virus and inform the development of effective diagnostic and control strategies (https://etd.aau.edu.et).

## 4. Discussion

The present study aimed to investigate an outbreak of lumpy skin disease (LSD) in cattle in the study area using multiple diagnostic methods, including clinical diagnosis, PCR, real-time PCR, and virus isolation. Out of the 73 typical clinical cases sampled and tested in the study, 47 and 49 were confirmed to be positive for LSD using PCR and real-time PCR, respectively. The use of multiple diagnostic methods allowed for the rapid and accurate detection of LSDV in the study samples, which is important for the early detection and control of the disease. The clinical signs found in the current study, such as circumscribed nodules on the skin, necrotic nodules, enlargement of superficial lymph nodes, and lacrimation, are in agreement with the findings of Alemayehu et al. [18]; Gari et al. [3]; and Gelaye et al. [5] in different areas of the country.

In the current study of LSD in cattle, there were 625 animals at risk; the overall morbidity, mortality, and case fatality rate were 11.68%, 1.92%, and 16.44%, respectively. In the current study, the morbidity rate (11.68%) is higher than that reported by Alemayehu et al. [19], who reported 6.1%. The morbidity rate in the present report was lower than the report of Leliso et al. [20]; which report 18%. Other studies reported wide ranges of morbidity rates in cattle, ranging from 3% up to 85%; Tuppurainen and Oura [6]. Furthermore, it is slightly higher than that reported in Davies [1] which showed that the usual morbidity rate is a range between 1 and 5%. The morbidity rate varies, especially when the outbreak of the disease rises, it depends on the susceptibility of cattle and the abundance and occurrence of vectors (arthropod vectors) that transmit the virus.

The present finding indicates the mortality rate (1.92%) which is slightly lower than that reported by Ayelet et al. [8] which reported 4.97%. The present finding agrees with the reports of Alemayehu et al. [18] and Leliso et al. [20] which recorded 1.8% and 1.34%, respectively. The present study indicated that the case fatality rate (16.44%) is lower than the reports of Ayelet et al. [8] and Alemayehu et al. [19]; who report 36.49% and 30% respectively. Moreover the present finding of case fatality rate was higher than the finding of Leliso et al. [20] who report 7.44%. However; many reports showed the morbidity and mortality rates of LSD vary. The morbidity rate of the disease ranges from 5 to 100% [21]. While occasional mortality rates of 10 to 40% have been reported, but the rate of 1 to 5% is usually observed [21]. These values differ based on a number of factors like geography, climate, management conditions, and immune status of the animals, breed and strain of virus involved, and the number and types of insect vectors [6].

In the current study, the lumpy skin disease virus was isolated by inoculation on cell culture (Vero cell) from collected samples. Characteristic pock lesions were observed after the 3^rd^ passage; this finding agrees with the study of El-Kenawy and El-Tholoth [7] that successfully cultivated LSDV to detect the characteristic pock lesions. The CPEs characterized by rounding of cells, aggregation of dead cells, and destruction of monolayers are in line with the reports made [8].

Conventional PCR and highly resolution melting real-time PCR have led to many major scientific advances. Though both methods are still regularly used in laboratories, real-time PCR is gaining popularity and quickly becoming the most cost- and time-effective method for analyzing DNA products. The conventional PCR assay used in this study identified a suitable target in the p32 genome. It showed high specificity as a unique band of the expected size of 192 bp obtained for DNA samples derived from skin lesions. Out of 73 skin biopsy samples, 47 (64.4%) samples were positive for lumpy skin disease in cattle. The present study was higher than the report of Ateya et al. [22] who isolated LSDV (21.7%) from CAM and reported similar pox lesions of LSDV.

LSDV was isolated on a Vero cell, and viral isolates were identified using RT-PCR and gel-based PCR to detect LSDV-DNA in skin scrap samples. The real-time PCR assay is for detecting viral DNA in skin biopsies. Out of 73 skin scrap samples, 67.1% (49/73) were positive by real-time PCR. Hence, real-time PCR was more sensitive to detecting skin scrap samples than conventional PCR. The real-time PCR assay is also a useful tool for early detection and control of infections by other CaPV viruses. The real-time PCR results showed proper efficacy in detecting viral DNA, especially in skin biopsies, which contain more viral particles, as mentioned before [22, 23].

In conclusion, the present study provides important insights into the occurrence, epidemiology, and diagnosis of LSD in the study area. The use of multiple diagnostic methods, including PCR, real-time PCR, and virus isolation, allowed for the rapid and accurate detection of LSDV in the study samples. The high positivity rates observed in the PCR and real-time PCR assays highlight the importance of early and accurate diagnosis for effective control of the disease. The results of this study can help inform the development of effective control strategies for LSD, which is a significant threat to cattle in the region.

## 5. Conclusion and Recommendation

Lumpy skin disease (LSD) poses a significant health concern for cattle in the research region, leading to substantial economic losses due to detrimental effects such as irreversible hide damage, reduced weight gain, diminished milk production, infertility, and miscarriages among pregnant cows. The ineffectiveness of the current LSDV vaccine in dairy cattle following vaccination has raised concerns regarding the necessity to reassess the existing vaccine strategy and policy.

In large-scale and high-intensity production systems, the primary routes of transmission for lumpy skin disease (LSD) are unrestricted cattle movement and direct or indirect contact among animals during grazing, drinking, and trading activities. Consequently, it is crucial for the government to establish effective control and eradication strategies, as well as implement awareness campaigns, to enhance the drainage system and regulate vector reproduction.

The findings of this study provide valuable insights that contribute to a better understanding of lumpy skin disease (LSD) and can be utilized to improve the management of Capripoxvirus infections. Further research is recommended to investigate the underlying reasons for the failure of the current immunization policy and to develop more effective strategies for LSD control, aiming to mitigate the significant economic burden associated with the disease.

## Figures and Tables

**Table 1:** The specific primers and probe used in this study.

Name	Primers (5′–3′)	Size of probe
LSDV-F	TGAATTAGTGTTGTTTCTTC	59 bp
LSDV-R	GGGAATCCTCAAGATAGTTCG	
LSDV-P	FAM-TGCCGCAAAATGTCGA-MGB	

**Table 2:** The morbidity, mortality rate, and case fatality rate of affected cattle in the study area.

District	No. of susceptible	No. of affected cattle	No. of death	Morbidity rate (%)	Mortality rate (%)	Case fatality rate (%)
Daaroo Labuu	235	31	5	13.19	2.13	16.13
Hawwii Guddinaa	189	29	3	15.34	1.59	10.34
Gumbii Bordode	201	12	4	5.97	1.99	33.33
Total	625	73	12	11.68	1.92	16.44

## Data Availability

The corresponding author can provide access to the datasets utilized and/or examined in the present study upon a reasonable request.
